# Prevalence and distribution of exposure to Schmallenberg virus in Irish cattle during October 2012 to November 2013

**DOI:** 10.1186/s12917-015-0564-9

**Published:** 2015-10-20

**Authors:** D. Barrett, S. J. More, R. O’Neill, B. Bradshaw, M. Casey, M. Keane, G. McGrath, D. Sammin

**Affiliations:** Department of Agriculture, Food and the Marine, Sligo Regional Veterinary Laboratory, Doonally, Sligo Ireland; Centre for Veterinary Epidemiology and Risk Analysis, UCD School of Veterinary Medicine, University College Dublin, Dublin 4, Ireland; Department of Agriculture, Food and the Marine, Central Veterinary Laboratory, Backweston Laboratory Complex, Celbridge, Co. Kildare Ireland; Department of Agriculture, Food and the Marine, Cork Blood Testing Laboratory, Model, Farm Road, Cork, Ireland

**Keywords:** Schmallenberg virus, Surveillance, Pathology, Serology, Prevalence, Ireland

## Abstract

**Background:**

Schmallenberg virus (SBV) was first identified in November 2011. It is a novel *Orthobunyavirus* (family *Bunyaviridae*) whose main ill effect is congenital malformation of the musculoskeletal and central nervous systems. It is borne by *Culicoides* spp., and has spread extensively in western Europe. The first case of SBV in Ireland was diagnosed in October 2012. It was anticipated that once the virus emerged in Ireland that there would be wide scale or nationwide spread over the course of the 2013 vector season. The objectives of this study were to determine the seroprevalence and distribution of exposure to Schmallenberg virus in Irish cattle from November 2012 to November 2013.

**Methods:**

Samples of brain for the pathology based surveillance were collected from malformed bovine and ovine foetuses submitted for post mortem examination. These samples were tested for SBV using RT-qPCR. Three serological surveys were carried out on sera submitted for the national brucellosis eradicartion programme. A spatial analysis of both sets of data was carried out.

**Results:**

Between October 2012 and 10th May 2013, SBV was confirmed by RT-qPCR in brain tissues from malformed foetuses obtained from 49 cattle herds and 30 sheep flocks in Ireland. In national serosurveys conducted between November 2012 until November 2013 the herd-level and animal-level SBV seroprevalences in cattle were 53 and 36 % respectively for the first survey, 51 and 35 % for the second survey and 53 and 33 % for the third survey. The herd level seroprevalence in counties ranged from 0 to 100 %, with the counties in the south and southeast having the highest seroprevalence (>50 %), the midlands a moderate herd level seroprevalence (10–50 %) while northern and north western counties had a low herd level seroprevalence (0–10 %). There was close spatial agreement between the results of the two different targeted surveillance strategies.

**Conclusions:**

At the end of the 2012 vector season, there was widespread exposure to SBV among herds in southern and south eastern Ireland. During 2013, there was little or no evidence of further outward spread, unlike the situation in several other European countries. Given the lack of evidence for circulation of the virus since 2012, it is likely that the younger age cohort in herds previously exposed to SBV and substantial proportions of animals of all ages on the margins of affected areas are immunologically naïve to SBV, and would be susceptible to infection if the virus were to re-emerge.

## Background

Schmallenberg virus (SBV), a novel *Orthobunyavirus* (family *Bunyaviridae*), was first identified by metagenomic analysis at the Friedrich Loeffler Institute in November 2011 [1]. The virus has since been detected in a variety of ruminant species including goats, deer and camelids, but cattle and sheep are considered the most important hosts [1]. SBV generally causes a transient non-fatal infection in adult ruminants. Infected adult ruminants may exhibit no observable clinical signs of the infection, or in the case of lactating dairy cows may show a non-specific, mild clinical syndrome (fever, diarrhoea, reduced milk production) for a few days [1]. The main effect of SBV arises from in utero infection of ruminant foetuses, resulting in congenital malformations of the musculoskeletal and central nervous systems.

Between December 2011 and January 2013, abortions, stillbirths and the birth of malformed lambs, calves and kids linked to SBV infection, and seroconversion to SBV, were reported throughout continental Europe [2]. Almost complete national seroconversion to SBV over a 12 month period was demonstrated in Belgium [3], the Netherlands [4] and Switzerland [5]. Both Dutch and Swiss serology studies appear to show rapid and almost uniform transmission of SBV features consistent with an aerial vector-borne disease. In one German herd, 100 % seroconversion was recorded over the space of 1 month [6]. SBV seroprevalence in one herd in southern England increased from 1.7 % in May 2012 to 89.1 % in November 2012 [7]. In the UK, the greatest prevalence of confirmed SBV cases was confined to the south and the south east of the country [8]. In France, seroconversion to SBV was first documented in October 2011 [9]. Significant regional differences in herd level seroconversion have been described in France, with highest levels up to 80 % along the eastern border with Germany, where the disease first emerged, falling to 10 % in the south west of the country [10]. Almost 75 % of bulk milk samples collected randomly over a 6 month period in Sweden during 2012 was found to be positive for SBV antibody [11], with malformed lambs and calves born in November 2012 and January 2013, respectively.

Vector transmission is considered the most significant transmission route for SBV, and a variety of *Culicoides* species are known to carry the disease [12]. Regional factors such as husbandry practices, stocking densities, land use, local vector populations and meteorological conditions are considered to account for the regional differences in infection pressure [13]. A Scottish model of SBV spread, in climatic conditions similar to Ireland, found that SBV posed a risk to livestock in an average year, and that in warmer than average (but feasible) conditions SBV could spread rapidly [14]. The purchase of a viraemic animal from an endemically infected region is a possible route of introduction. The method and timing of the introduction of SBV to Ireland are the subject of parallel research and beyond the scope of this paper.

While a number of tick-borne diseases are endemic in Ireland, SBV-related teratogenesis presents as the first disease entity to have been transmitted to Irish ruminants by an aerial arthropod vector. The first confirmed clinical case of SBV malformation was diagnosed in Ireland in a bovine foetus in a herd in Co. Cork in October 2012 [15]. There have been no confirmed incidents of SBV-associated clinical syndrome of fever, milk drop and acute diarrhoea in adult cattle in Ireland. Regional variations in the spread of SBV infection were documented in the UK and France [10], and underreporting of clinical SBV is well recognised [9]. Consequently, a series of three structured surveys were carried out to provide additional evidence of the exposure of the cattle population to Schmallenberg virus infection.

The objectives of this study were to determine the prevalence and distribution of seroconversion to Schmallenberg virus in Irish cattle at the end of the 2012 vector season, to establish what if any further spread occurred during the 2013 vector season and to compare the spatial distribution of confirmed cases of SBV (malformed foetuses) with that of seroconversion to SBV.

## Methods

### Pathology-based surveillance

Pathology-based surveillance for diseases of Irish farmed animals is delivered by a strategically located network of six regional veterinary laboratories (RVLs) operated by the Department of Agriculture Food and the Marine (DAFM). Virtually all of the submissions (carcasses and clinical pathology samples) are made voluntarily by herd/flock owners on referral by the attending veterinary practitioners. In order to ensure early detection of SBV incursion into Ireland, an information campaign was launched in early 2012 to increase awareness of the disease and to incentivise submissions; post mortem examination of malformed foetuses in RVLs was provided to farmers free of charge. Samples of brain from cases of abortion or stillbirth in bovine and ovine foetuses with lesions suggestive of SBV such as hydranencephaly or arthrogryposis were tested using a real-time reverse transcription PCR (RT-qPCR) detecting an 88 bp fragment of the S3-segment of the SBV genome, as developed at the Friedrich-Loeffler Institute [16].

### Serological surveillance

Three national serological surveys were conducted to estimate SBV exposure in Irish cattle, using sera collected for the national brucellosis eradication programme between November 2012 and November 2013. For the national brucellosis eradication programme, 20 % of cattle herds in Ireland were tested in each of 2012 and 2013, different herds being tested in each year. These herds were randomly distributed throughout the country and in each of these herds blood was collected from all breeding cattle that were 2 years of age and older. For each SBV serological survey, at least 17 herds were selected from each county (there are 26 counties in Ireland) and six animals from each herd. This was based on a presumed within-herd prevalence of 70 % and herd-level prevalence of 1 % in order to estimate the county-level prevalence with a 95 % confidence interval and a precision of +/−5 %. In those counties with larger cattle populations more than 17 herds were selected on each occasion such that a national total of 532 herds were included in each survey (442 herds was the number required to estimate national herd level prevalence with 95 % confidence and a precision of +/−1 %). Herd enrolment per county was conducted sequentially, according to the order of sample processing at the Cork Blood Testing Laboratory, until sufficient herds were enrolled in each county. Six samples were tested in each enrolled herd, usually the first six within each herd submission. The animals from the first survey were sampled between November 2012 and January 2013, apart from County Dublin where it extended to March 2013. The second survey was conducted between June and August 2013. The third survey was conducted between October and November 2013.

Sera were screened using a commercially available indirect SBV Antibody Kit (Part No. 99-41259; Idexx Laboratories) without modification. A sample/positive (S/P) ratio of 40 % was the cut-off for determining positive serum samples. Samples with an S/P of less than 30 % were considered negative and samples with an S/P greater than 30 % and less than 40 % were considered suspect. The manufacturer reports the sensitivity to be 98.1 %, while the specificity is estimated at 99.5 % [17].

#### Ethical statement on sample collection

During this study, two types of tissue specimen were collected. Brain tissue was collected from aborted (dead) foetuses as part of a post mortem diagnostic protocol. Serum was obtained from blood samples which were collected as part of the national brucellosis eradication programme. From an ethical perspective, all of the material collected and used as part of this study was outside the scope of Directive 2010/63.

### Data management and analysis

Data management and analysis was conducted using Excel 2007 (Microsoft Corporation, Redmond, WA, USA) and ArcGIS 10.1 (ESRI, Redlands, CA, USA). A herd was considered serologically positive if there was at least one seropositive among those animals sampled from that herd. All Irish herds have a GIS grid reference, which is based on the centre point of the largest parcel of land associated with the herd in question. This GIS grid reference was used for each of the herds that were sampled and tested in this study. To analyse the spatial distribution of pathologically positive case herds and seropositive herds, a 50x50km square grid was laid over maps of serological and pathological results. The pathological herd incidence and the serological herd prevalence were calculated for each square grid. The pathology herd incidence per square grid was calculated by dividing the number of herds where SBV was confirmed in the square grid by the total number of herds in that square grid where a suspect foetus was tested for SBV. The serological herd prevalence per square grid was calculated by dividing the number of herd with one or more seropostive animals by the number of herds tested in that square grid. The spatial correlation between the results of the pathological and serological surveillance strategies was assessed using a Spearman’s rank correlation.

## Results

### Pathology-based surveillance

**Fig. 1:**
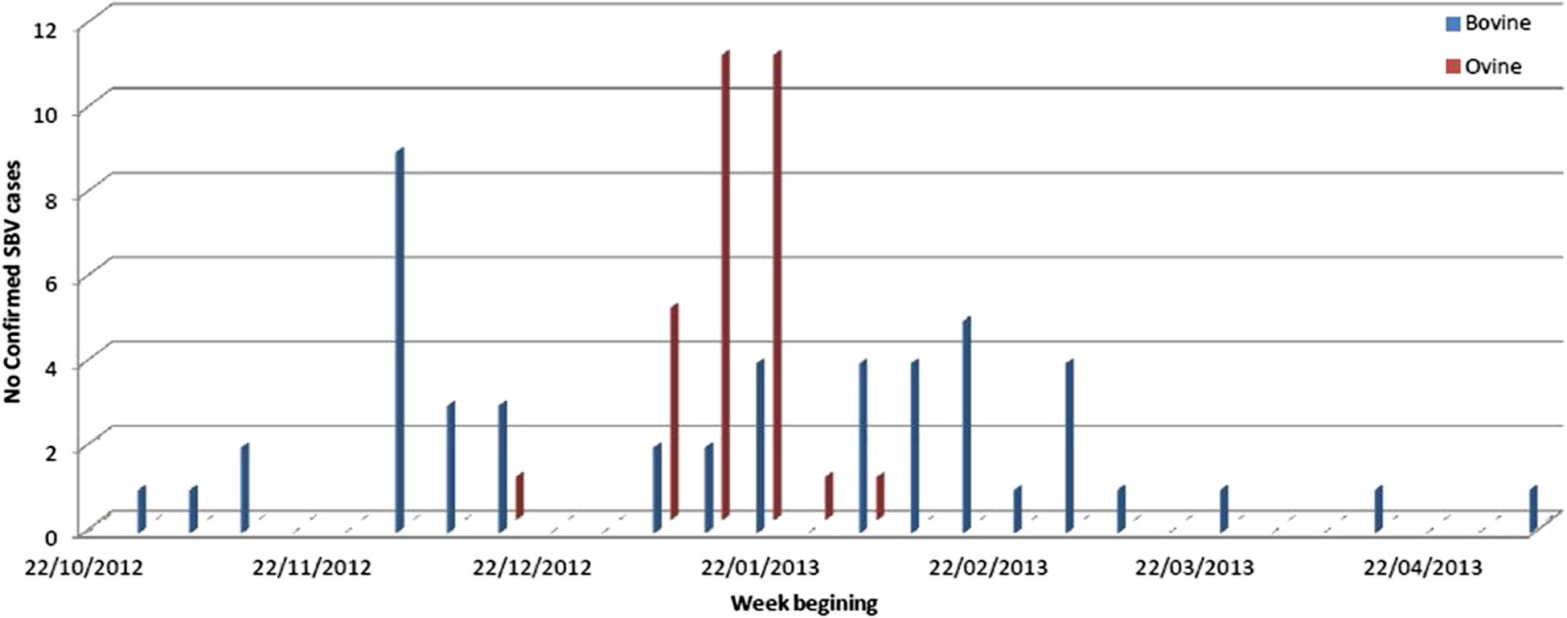
Chronology of confirmed cases of SBV

**Fig. 2:**
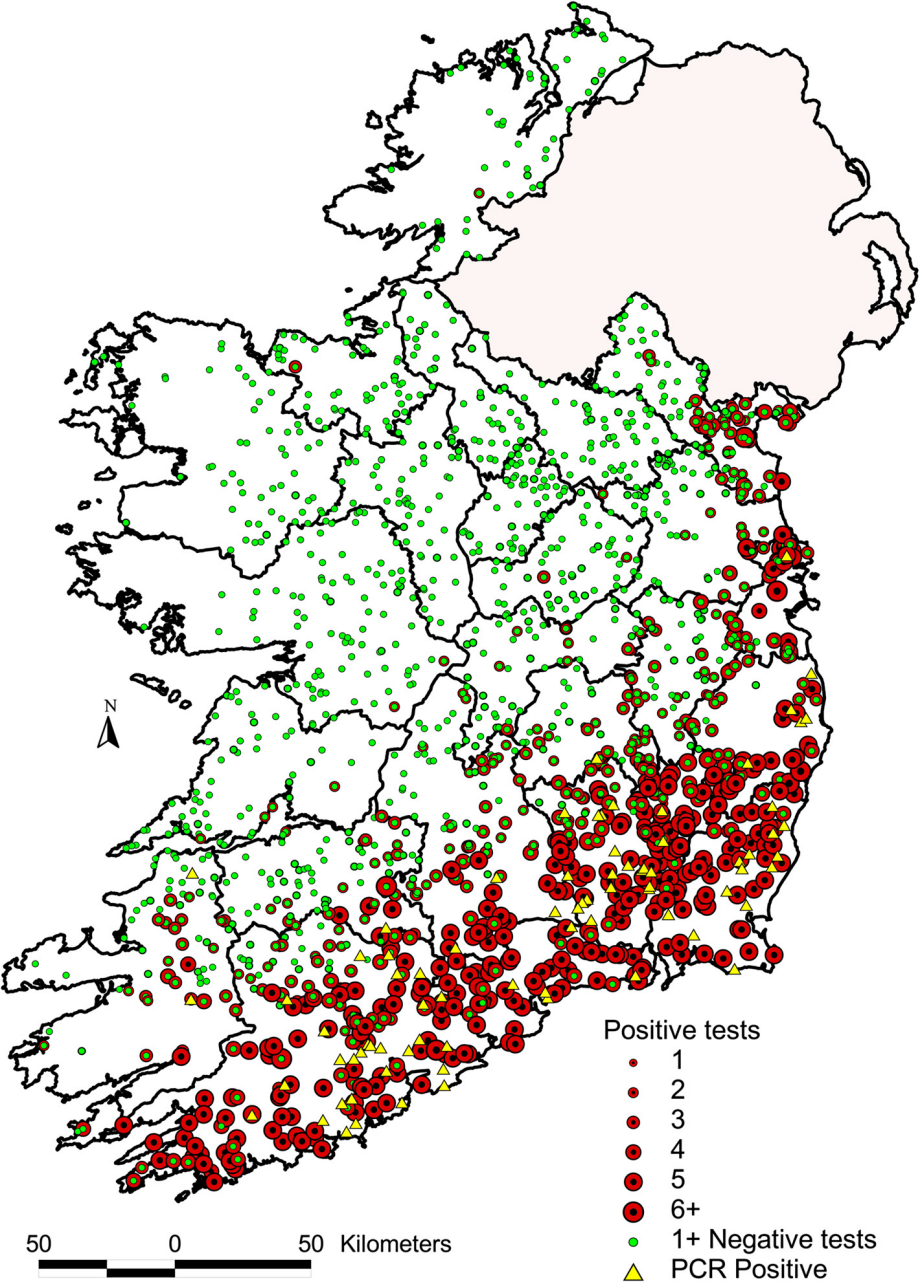
Spatial distribution of samples collected through pathology based and serological based SBV surveillance in Ireland during 2012 and 2013. The location of RT-qPCR-positive animals, which were submitted to veterinary laboratories between 30 October 2012 and 9 May 2013, is presented as *yellow triangles*. The *green* and *red dots* highlight the location of farms enrolled in the 3 serological surveys during November 2012 to November 2013, with colour and size relating to the number of animals per farm that were positive to SBV

Between October 2012 and May 2013, SBV was confirmed by RT-qPCR in brain tissues from malformed foetuses obtained from 49 cattle herds and 30 sheep flocks in Ireland (Table 1 and Fig. 1). These herds and flocks were geographically distributed in the south, south east and east of the country (Figs. 1, 2).

**Table 1:** The number of SBV RT-qPCR positive herds in Ireland, during 2012/13

County	Total	No Bovine cases	Susceptible period in utero for bovine cases ^a^	No ovine cases	Susceptible period in utero for ovine cases ^a^
Cork	28	23	25th Mar to 8th Dec	5	9th Sept to 27th Oct
Wexford	13	5	22nd Apr to 17th Nov	9	19th August to 27th Oct
Kilkenny	19	9	8th May to 8th Jan	9	9th Sept to 3rd Nov
Tipperary	3	1	8th May to 17th Nov	2	15th Sept to 27th Oct
Waterford	5	4	3rd Jun to 6th Oct	1	15th Sept to 27th Oct
Kerry	2	2	1st July to 29th Dec		
Wicklow	5	2	16th Jun to 6th Oct	3	15th Sept to 27th Oct
Carlow	2	1	9th July to 25th Oct	1	15th Sept to 15th Oct
Limerick	1	1	9th July to 25th Oct		
Laois	1	1	9th July to 25th Oct		
	79	49		30	

^a^The national bluetongue survey revealed that although midge activity occurred between April and December each year, there was minimal midge activity after the end of October each year [22]

### Serological surveillance

**Fig. 3:**
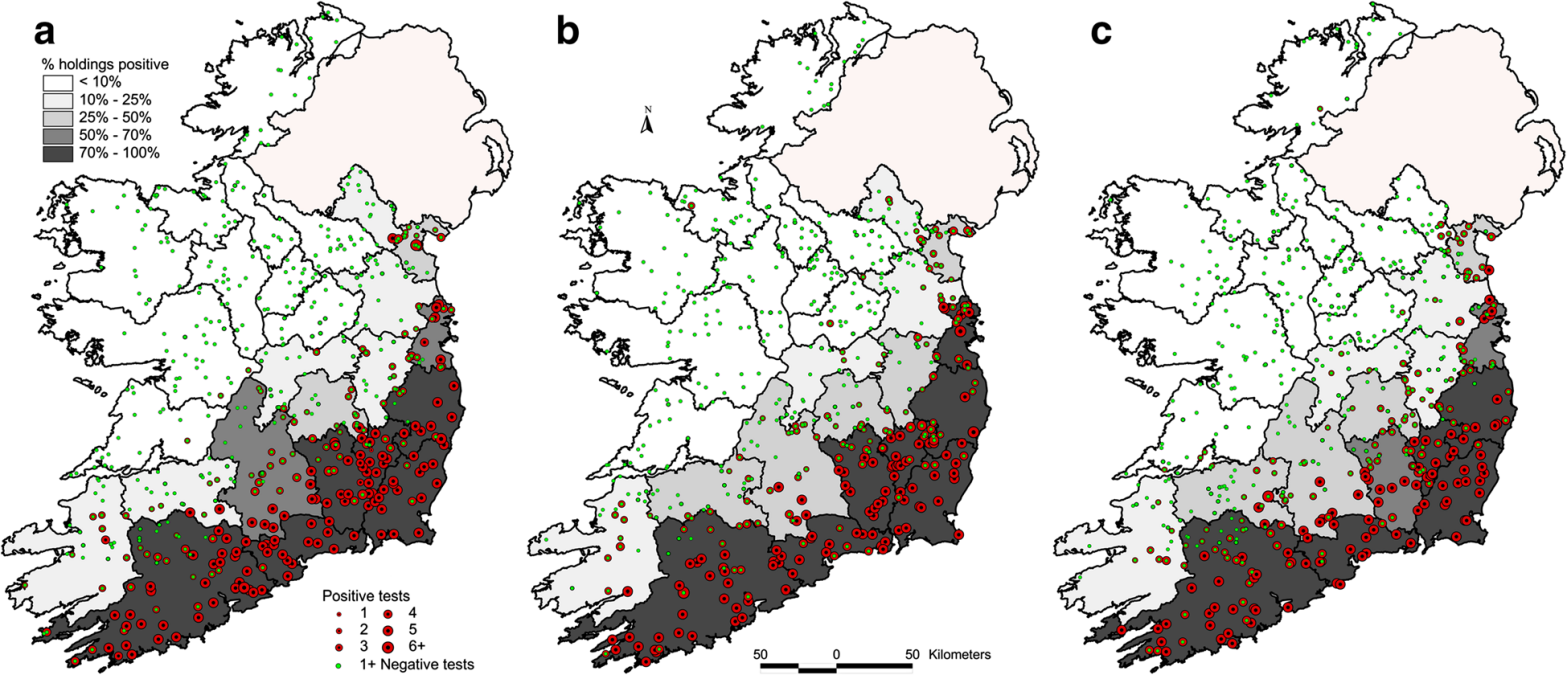
County-level herd SBV prevalence, and the spatial distribution of herds positive to SBV, based on active surveillance. The colour and size of each *dot* refers to the number of animals per farm that were positive to SBV. **a** During November 2012–January 2013. **b** During June–August 2013. **c** During October–November 2013

At the first, second and third SBV surveys, samples were collected from 3192 cattle from 529 herds, 3101 samples from 517 herds, and 3204 samples from 534 herds, respectively. The herd-level and animal-level SBV seroprevalences were 53 and 36 % respectively for the first survey, 51 and 35 % for the second survey and 53 and 33 % for the third survey (Table 2). There was no significant difference between these prevalences (*p* = 0.12). The herd-level seroprevalence within counties ranged from 0 to 100 %, with the counties in the south and southeast having the highest seroprevalences (>50 %), those in the midlands have moderate herd-level seroprevalences (10–50 %) while northern and north-western counties had low herd-level seroprevalences (0–10 %) (Fig. 3).

**Table 2:** Animal and herd SBV seroprevalence in Irish cattle during 2012/13, by province and county

	Survey 1 (Nov 2012–Jan 2013) ^a^				Survey 2 (Jun–Aug 2013)				Survey 3 (Oct–Nov 2013)			
	Animals		Herds		Animals		Herds		Animals		Herds	
Province and County	Total	% positive	Total	% positive	Total	% positive	Total	% positive	Total	% positive	Total	% positive
Connacht												
Galway	168	3.6	28	14	168	0	28	0	168	1.8	28	7
Leitrim	102	2	17	12	102	2.9	17	18	102	2	17	12
Mayo	114	1.8	19	11	114	1.8	19	10	114	0.9	19	0
Roscommon	102	0	17	0	102	2	17	12	102	2.9	17	18
Sligo	102	1	17	6	102	0	17	0	102	2	17	12
Leinster												
Carlow	102	96.1	18	100	102	95.5	17	100	102	82.4	17	100
Dublin	102	63.7	16	88	102	83.3	17	100	114	53.5	19	94
Kildare	102	20.6	16	63	102	27.5	17	82	102	21.6	17	65
Kilkenny	126	84.9	21	100	126	80.2	21	100	126	69	21	95
Laois	102	36.3	17	71	102	25.5	17	65	102	27.5	17	76
Longford	102	1	17	6	102	2	17	12	102	4.9	17	29
Louth	102	37.3	16	75	102	39.2	17	82	102	42.2	17	88
Meath	102	11.8	17	35	102	18.6	17	41	102	17.6	17	59
Offaly	102	14.7	17	59	102	13.7	18	44	102	10.8	17	41
Westmeath	102	5.9	17	36	102	5.9	17	23	102	1	17	6
Wexford	102	97.1	17	100	102	96.1	17	100	102	98	17	100
Wicklow	102	74.5	16	88	102	72.5	17	100	102	73.5	17	100
Munster												
Clare	114	2.6	19	11	114	1.8	19	11	114	8.8	19	37
Cork	354	77.1	59	95	263	88.2	43	98	354	70.3	59	88
Kerry	138	21	23	52	138	23.9	23	52	138	18.8	23	57
Limerick	156	20.5	26	58	156	26.3	26	73	156	26.9	26	58
Tipperary	186	55.4	31	77	186	39.2	31	61	186	39.2	31	68
Waterford	102	99	17	100	102	94.1	17	100	102	97.1	17	100
Ulster												
Cavan	102	2	17	12	102	2	17	12	102	2	17	12
Donegal	102	0	17	0	102	2	17	12	102	5.9	17	23
Monaghan	102	12.7	17	24	102	12.7	17	29	102	9.8	17	29
Total	3192	35.8	529	53.3	3101	35.2	517	51.4	3204	33.2	534	52.8

^a^In Dublin, samples were collected during February and March 2013

### Spatial analysis

The pathological and serological herd prevalences for each square grid are outlined in Fig. 3. The highest pathological herd incidence was recorded in the most south eastern square grid, and the two square grids north of it. The highest serological herd prevalence covered a wider area over the south and south east. The square grids where the pathological surveillance system had identified SBV generally had the greatest seroconversion to SBV. The Spearman’s rank correlation on the grid square values of seroprevalence to pathology-prevalence was 0.6133, indicating a correlation between the two surveillance methodologies.

## Discussion

SBV seroconversion was greatest in the south and south east of Ireland. In the initial 2012 survey, 100 % herd-level seroprevalence was recorded in four counties and >75 % in a further five counties within this region. In the former four counties, animal-level seroprevalence ranged from 85 to 99 %. Both animal and herd-level seroprevalences tended to diminish in the more north western parts of the country (Fig. 3a). Factors such as local vector populations, vector species competence, topography and ambient temperature are known to influence the infection rate among farm animal species. Midge biting rate, extrinsic incubation period and vector mortality rates are all known to be temperature dependent, and all influence the transmission of a vector borne disease [18]. The availability of suitable vector habitats and suitable hosts and the characteristics of the pathogen are key determinants in facilitating the transmission of a vector born disease [19]. One possible explanation as to why seroconversion was confined to the south and east was that SBV had been introduced relatively late in the vector season. The limited geographic extent of spread of SBV in Ireland at the end of 2012 may be attributable to the timing of incursion, which most probably occurred in the latter half of the vector active season. At the end of the first vector season after SBV was introduced into England, a similar pattern was evident in that seropositive sheep flocks and clinical cases were confined to the south and east of the country [8, 18], whereas in the Netherlands, herd prevalences in all regions ranged from 90 to 100 % at the end of the first vector season [4]. A Scottish model has shown that SBV introduction late in the vector season in climatic conditions similar to Ireland markedly reduces the spread of infection compared to an introduction earlier in the vector season [14]. The reasons for the reduced transmission include the shorter duration of the remaining vector active period, the bimodal distribution of *Culicoides* activity, and the lower temperature later in the vector season.

There was little evidence of further spread of SBV over the course of the second vector season after viral introduction (Fig. 2a, b, c). Indeed, the results of the serological surveys conducted during the latter half of summer 2013 and late autumn 2013 were little different from those obtained from the initial 2012 serosurvey. Based on the experience in several other European countries, it had been anticipated that SBV transmission would resume with the commencement of the 2013 vector active season, leading to an extension of the geographic distribution of exposed herds in Ireland towards the north and west. However, this anticipated spread never materialised, and suggested reasons for this relate to adverse weather conditions in the spring of 2013, the virus failing to reactivate after the winter of 2012 and herd immunity. The spring and early summer of 2013 were very cold, wet and windy in Ireland [20], which is likely to have delayed the resumption of midge activity [22]. SBV has successfully overwintered, despite lengthy periods of minimal vector activity [21]. Data from Germany has shown that midge activity during cold weather is minimal [23]. Another possible explanation for the lack of spread in 2013 is “herd immunity” attributable either to previous exposure to the virus or the use of SBV vaccines. The high rate of seroconversion achieved in the first vector season in affected counties would have resulted in a high frequency of immunity in the second season [24]. If there was a further incursion of SBV in 2013 from either the UK, or mainland Europe, into a previously infected region, transmission could be limited due to widespread pre-existing immunity. It is unlikely that vaccination had any significant impact as vaccine uptake among Irish cattle farmers was low, because the vaccine only became available after the end of the spring breeding season (and the second serosurvey presented here) and could not have affected seroconversion in the first and second serosurveys.

The remote geographical location of Ireland is one of the most important factor in limiting the incursion and spread of vector-borne viral diseases. For example, while Bluetongue spread rapidly and widely throughout the ruminant populations of north-western Europe in 2006/2007, including into the south-east of England [25] there was no evidence of incursion into Ireland during that outbreak. Ireland’s remote location means complex rare meteorological events are likely to be required to facilitate the wind borne introduction of a vector borne disease into the country. A Scottish SBV model indicates that introduction of SBV must occur relatively early in the vector season to bring about extensive spread [14]. The model also showed that mean Scottish summer temperatures (similar to Irish summer temperatures) facilitate only limited spread as SBV is so temperature dependent. Assuming this model is applicable to Ireland, for extensive spread of SBV by a wind borne vector to occur requires a relatively rare meteorological event to occur early in the vector season in a year with above average temperatures. While these events can occur, the fact that all three are necessary reduces the probability of SBV being successfully established in Ireland.

**Fig. 4:**
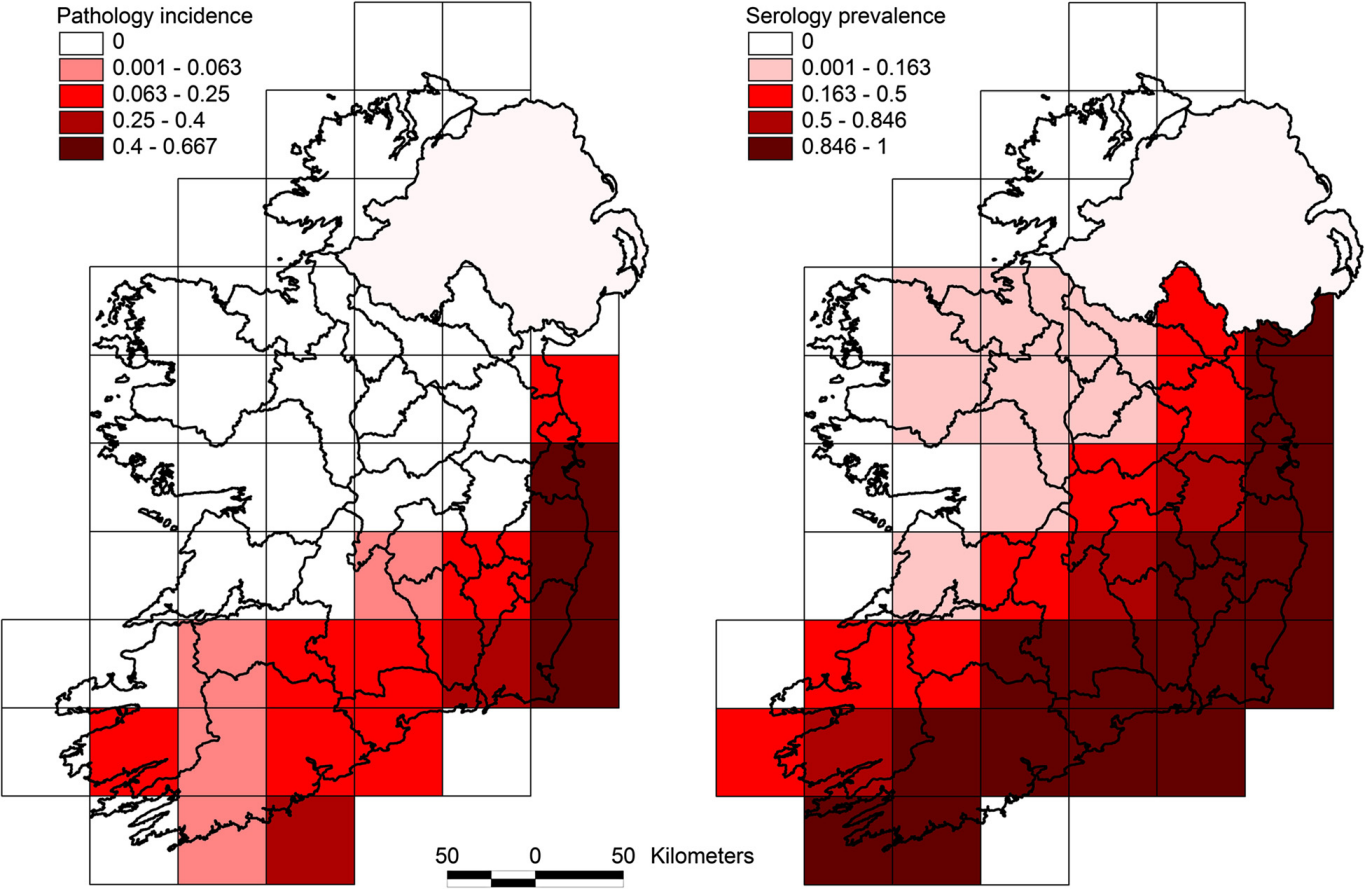
Pathological herd incidence and serological herd prevalence per 50 × 50 km square grid

The spatial distribution of SBV seroconversion was correlated with the spatial distribution of confirmed SBV cases in malformed ovine and bovine foetuses (Fig. 4). The pathological surveillance strategy was active and risk-based, with the objective of detecting cases, from a biased set of samples where SBV was suspected at gross post mortem examination. While the serological based surveillance was also active, it was not risk based, but was designed with the objective of estimating prevalence and/or proving freedom from infection from a truly random sample of the population. The pathology-based surveillance had correctly identified the regions most affected by SBV (Fig. 3). The key concern for pathological based surveillance is the ability to detect cases of SBV malformation, while the purpose of the serological surveillance was to provide an accurate quantification of the extent of SBV exposure to be extrapolated nationally. While the two strategies were complimentary, they were not directly comparable.

The serological sampling strategy involved the opportunistic real-time selection of serum samples submitted for the national brucellosis serological surveillance programme, as this was considered the most cost effective and efficient way to access serum from cattle throughout the country. The parameters set out in the sampling plan were conservative from a herd-level perspective. In designing the sampling frame, it was assumed that if SBV was present in a herd, there would be relatively high within-herd prevalence as had been indicated by serological data from several European countries. The within herd prevalence was set at 70 % which is relatively low compared to the levels of seroconversion seen in exposed herds in other countries. The herd level prevalence was set at 1 % to detect a low level herd prevalence. While there is always the potential for bias with an opportunistic sampling strategy, this was addressed in part by selecting herds which had already been randomly selected for the national brucellosis eradication programme. The consistency in the results between the three serological surveys, in itself, suggests that there was limited bias in sample selection. Where single seropositive animals were identified in herds within those regions with low herd prevalence this may have been the result of moving animals that had already been exposed from the high prevalence areas of the south and south-east rather than local vector-borne spread. A previous study has shown there is considerable movement of cattle within Ireland [26]. In separate serological studies to assess SBV exposure in sheep flocks, follow-up of a single seropositive sheep in a county with low levels of seroconversion, showed that it had been purchased from the south east [27]. Such movements rather than “false positive” test results are a more likely explanation of singletons given the reported specificity of the serological test method that was used.

In future serological studies on SBV in Ireland, it would be prudent to sample animals born in or after 2013 in the high prevalence areas disclosed in this study, to determine if infection is present and active in those areas. Several studies have shown that cattle are more attractive to biting midges than other ruminant species, which makes them the ideal sentinel species for a midge borne disease. In one study 83 % of midges collected originated from cattle and 17 % originated from sheep, and almost 50 % of the midges originating from cattle were blood engorged versus 7 % of the midges which originated from sheep [28]. The present study has shown marked regional variation in SBV seroprevalences. Any future prevalence studies of vector borne disease could be informed by this gradient information and adjust/set their sampling frames on an intelligent regional basis rather than on a single national basis. It would be prudent to continue national serosurveys and the targeting of animals expected to be immunologically naïve, i.e. 2013 and 2014 born animals in the high prevalence areas in this study, to monitor SBV in the future. SBV is a relatively low impact disease, but shares a vector with pathogens that may potentially be of much greater significance. The patterns revealed and understanding gained with SBV should prove transferrable to other arboviruses. The epidemiology of SBV in its *Culicoides* vector is also worthy of further investigation in an Irish context. Understanding the ecology of various midge species and their competence as viral vectors will be very important in assessing the risk of viral introduction into and spread within Ireland in the face of any future midge-borne viral epizootic in Europe.

## Conclusion

The initial serologial survey carried out at the end of 2012 showed widespread exposure to SBV in southern and south eastern Ireland. Subsequent surveys in 2013 showed little or no evidence of any further outward spread. Unlike, several other European countries there was no evidence of spread in the second vector season. Given the lack of evidence for circulation since 2012, it is likely that younger age cohorts in herds previously exposed to SBV and animals of all ages in herds on the margins of affected areas are immunologically naive to SBV, and susceptible if the virus was to re-emerge. 
